# Tetrodotoxin Derivatization with a Newly Designed Boron Reagent Leads to Conventional Reversed-Phase Liquid Chromatography

**DOI:** 10.3390/toxins16060260

**Published:** 2024-06-04

**Authors:** Shimba Kawasue, Kyoko Kuniyoshi, Masashi Uema, Naomasa Oshiro

**Affiliations:** National Institute of Health Sciences, 3-25-26 Tonomachi, Kawasaki 210-9501, Japan; k-kuniyoshi@nihs.go.jp (K.K.); m-uema@nihs.go.jp (M.U.)

**Keywords:** tetrodotoxin, derivatization, reversed-phase HPLC-UV, boronic acid, pufferfish

## Abstract

Tetrodotoxin (TTX) is a representative natural toxin causing pufferfish food poisoning, which is especially prominent in East and Southeast Asia, including Japan. TTX has been analyzed through post-column derivatization high-performance liquid chromatography (HPLC), ion-pair LC-MS(/MS), and hydrophilic interaction liquid chromatography (HILIC)-MS(/MS) as alternatives to the mouse bioassay method. However, post-column derivatization requires a system for online derivatization reactions, and with the ion-pair LC-MS approach, it is difficult to remove residual ion-pair reagents remaining in the equipment. Moreover, HILIC-MS provides poor separation compared to reversed-phase (RP) HPLC and requires a long time to reach equilibration. Therefore, we decided to develop a TTX analytical method using pre-column derivatization and RP HPLC for the rapid assessment of outbreak samples, including food remnants. In this study, we focused on the *vic*-diol moiety of TTX and designed a new derivatization reagent coded as NBD-H-DAB. This NBD-H-DAB was synthesized from 4-hydrazino-7-nitro-2,1,3-benzoxadiazole (NBD-H) and 3-fluoro-2-formylphenylboronic acid (FFPBA) with a simple reaction system and rapidly converted to its boronate form, coded NBD-H-PBA, in an aqueous reaction solution. The NBD-H-PBA demonstrated appropriate hydrophobicity to be retained on the RP analytical column and successfully detected with a UV spectrometer. It was easily reacted with the *vic*-diol moiety of TTX (C6 and C11) to synthesized a boronic ester. The derivatized TTX could be detected using the RP HPLC-UV, and the limit of detection in the fish flesh samples was 0.06 mg/kg. This novel pre-column derivatization of TTX with NBD-H-PBA proves capable for the analysis of TTX.

## 1. Introduction

Tetrodotoxin (TTX) is the most historically recognized and studied marine biotoxin responsible for pufferfish food poisoning, which is especially prominent in the East and Southeast Asian regions [1,2]. From a food safety perspective, the analysis of TTX in food and fish specimens is essential. The mouse bioassay (MBA) method has previously been employed for TTX detection and toxicity quantification based on the symptoms in mice and the dose–death time relationship, respectively [1]. Nevertheless, regarding animal tests, an MBA has issues with selectivity, reproducibility, accuracy, and animal welfare. Therefore, it is necessary to develop alternative chemical analytical methods [3,4]. However, TTX’s chemical characteristics, including its highly polar nature and lack of a UV absorption group, has impeded the development of chemical analytical methods using the widely utilized reversed-phase (RP) high-performance liquid chromatography (HPLC) [5].

To date, three instrumental analytical methods for TTX have been developed. In the HPLC post-column derivatization method, TTX and its analogs (TTXs) are separated with a C30-RP column using an ion-pair reagent such as ammonium heptafluorobutyrate and the eluates are treated with a strongly basic condition at a high temperature (e.g., with 4 M NaOH at 105 °C) to generate a fluorescent C9 base [4,6]. This method allows the analysis of TTXs with high sensitivity and separation using a fluorescence detector. However, this post-column derivatization method requires specific instrument settings, and the processing and disposal of the resulting waste liquid is troublesome [3,4,6,7,8].

The ion-pair LC-MS method uses an ion-pair reagent (e.g., heptafluorobutyric acid) to solve the problem of analyzing TTX, similar to the HPLC post-column derivatization method [9,10,11,12,13]. However, the ion-pair reagents used for LC-MS can remain on the LC-MS line and analytical column after TTX analysis, when using the HPLC system for a different type of analysis, they might be carried over and interfere with other analyses.

Recently, the HILIC-MS method has been applied for TTX analysis [14,15,16,17,18,19,20]. HILIC columns are suitable for analyzing TTX because they can retain highly polar compounds. This method also allows it to elute under a high organic solvent ratio, allowing for a more sensitive analysis by LC-MS [21]. Therefore, HILIC-MS/MS is employed in most TTX analyses. However, some issues remain: e.g., this approach requires a long time for conditioning and analytical runs, broader peaks may decrease its sensitivity, and so on.

In this study, we focused on the esterification of boronic acid with *vic*-diol, which is easily completed under mild conditions. Here, we report an investigation into the design and synthesis of a novel pre-column derivatization reagent coded as NBD-H-DAB, bearing a UV absorption group and a functional group selectively reacting with the *vic*-diol moiety of TTX. We also study the established analytical methods for analyzing TTX using the conventional RP HPLC-UV system.

## 2. Results and Discussion

### 2.1. Design of Derivatization Reagent

**Figure 1:**
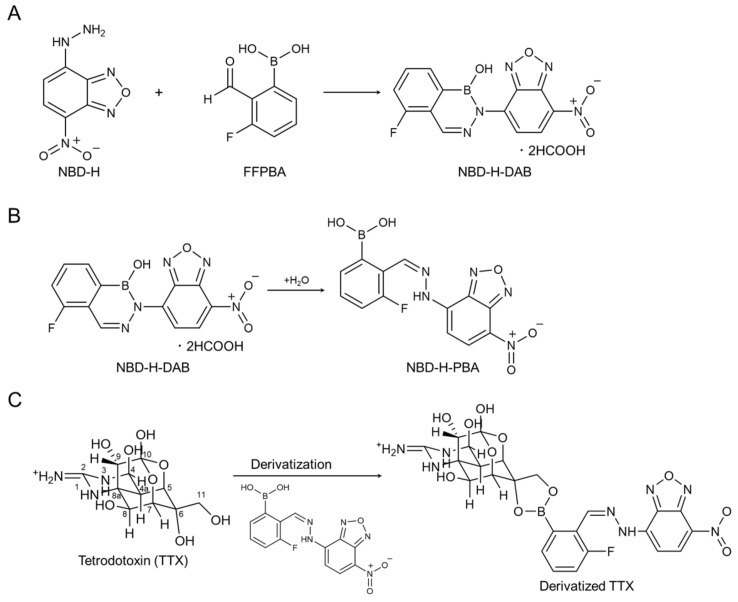
Synthesis of the derivatization reagent, NBD-H-DAB (**A**), its hydrolyzation under aqueous conditions, deriving NBD-H-PBA (**B**), and derivatization reaction of TTX with NBD-H-PBA (**C**).

The derivatization reagent was designed to react with the *vic*-diol moiety at C6 and C11 of TTX and to be capable of UV absorption on the long-wavelength side to reduce interference from the matrix components. The *vic*-diol moiety has been reported to react selectively with phenylboronic acid to form stable boronic esters, and this reaction has been used for the derivatization and purification of target compounds from complicated matrices in the past [22,23,24,25]. Boronic esters are stable in bulkier structures [26], and phenylboronic acids react at lower pH levels through increasing the acidity of the boronic acid moiety [27]. Therefore, a novel derivatization reagent, NBD-H-DAB (Figure 1A), was developed in this study and designated for TTX analyses. This NBD-H-DAB was synthesized by reacting 4-hydrazino-7-nitro-2,1,3-benzoxadiazole (NBD-H) with 3-fluoro-2-formylphenylboronic acid (FFPBA), which has an electron-withdrawing group (Figure 1B). The synthesized NBD-H-DAB was easily hydrolyzed under aqueous conditions to derive boronic acid, coded as NBD-H-PBA (Figure 1B) [28,29]. Thus, the derivatization reaction was performed under heating in water, NBD-H-DAB was rapidly converted to NBD-H-PBA and reacted with TTX (Figure 1C). In this study, the NBD-H-PBA was thought to have an imine-mediated 7-nitro-2,1,3-benzoxadiazole (NBD) structure at the *o*-position of the boronic acid, which not only increased the stability of the derivatized TTX, but also allowed it to react under weakly acidic conditions. NBD-H-PBA has an absorption maximum at 503 nm (Figure S3A), so the derivatized TTX also had this absorption maximum at the long-wavelength side. This reduced any interference from the matrix components in the fish flesh (Figure S3B).

### 2.2. Optimization of Derivatization Conditions

To investigate the optimal derivatization reaction conditions in the matrix, TTX-spiked fish flesh extracts were prepared and used. The derivatization conditions were optimized based on the HPLC-UV peak area of the derivatized TTX for various pH levels, solvent systems, concentrations of the derivatization reagents, temperatures, and reaction times. At the evaluated pH levels using acetate buffers (pH 3, 4, 5, and 6), the peak area of the derivatized TTX increased with the pH up to pH 5 but decreased at pH 6 (Figure S4A). The lower peak intensity observed at pH 6 may be due to the degradation of TTX since TTX is only a stable compound under weakly acidic conditions [30]. Although the basic condition is suitable for the esterification of boronic acid [27], the acidification of the boronic acid moiety might have improved the reactivity, as the boronic acid reacted well at pH 5.

Generally, conditions with less water are suitable for the esterification of boronic acid [22]. However, TTX is only soluble in acidic solutions, so we dissolved the derivatization reagent in 50–100% THF in water and compared the reactivity to produce a TTX derivative based on the peak area of the derivatized TTX. As a result, it was confirmed that the derivatization efficiency increased as the water ratio decreased. Therefore, the derivatization reagent was dissolved in 100% THF (Figure S4B). We also studied varying the concentration of NBD-H-DAB in the range of 0.1–3 mg/mL. As a result, the peak areas of the derivatized TTX became constant at values higher than 2 mg/mL (Figure S4C). The 2 mg/mL concentration of NBD-H-DAB was thus selected as the optimal concentration. Moreover, the reaction temperature (40, 50, and 60 °C) and time (5, 10, 30, 60, and 120 min) were varied, and the highest peak areas of the derivatized TTX were recorded at 50 °C, 60 min and at 40 °C, 120 min (Figure S4D). However, under high-temperature conditions, the TTX and derivatization reagents were at risk of decomposition. Therefore, the optimal reaction temperature and time were set at 40 °C and 120 min.

### 2.3. Effects of the Derivatization of TTX and Structural Analysis

**Figure 2:**
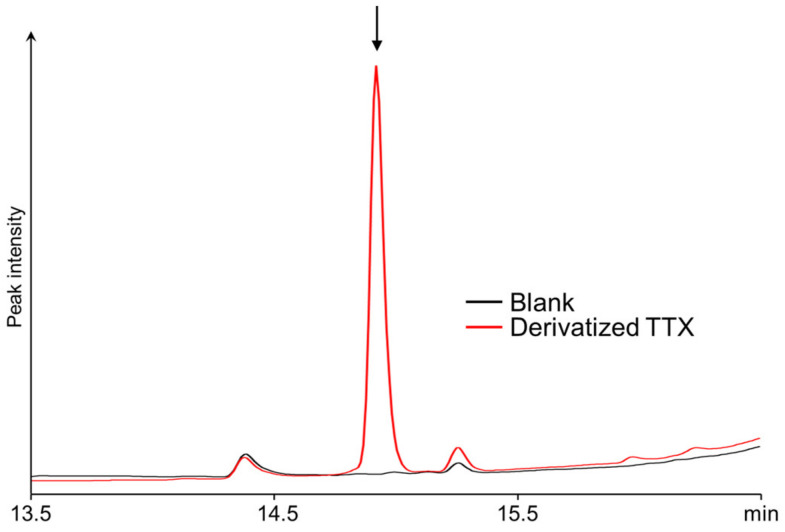
HPLC-UV chromatograms obtained after the derivatization reaction with NBD-H-DAB (NBD-H-PBA) of the solutions containing (red) or not containing (black) TTX.

Since the derivatization reaction of TTX with NBD-H-DAB was carried out under aqueous conditions, NBD-H-DAB was immediately hydrolyzed to produce NBD-H-PBA. Thus, the resulting product was a boronate ester of TTX and NBD-H-PBA. This derivatized product was analyzed using HPLC-UV with a C18 column. The peak suspected to correspond to the TTX derivative with NBD-H-PBA was detected at a retention time of 14.9 min (Figure 2). The compound was retained well on the RP column and could be analyzed using RP-HPLC coupled with a UV detector (510 nm).

**Figure 3:**
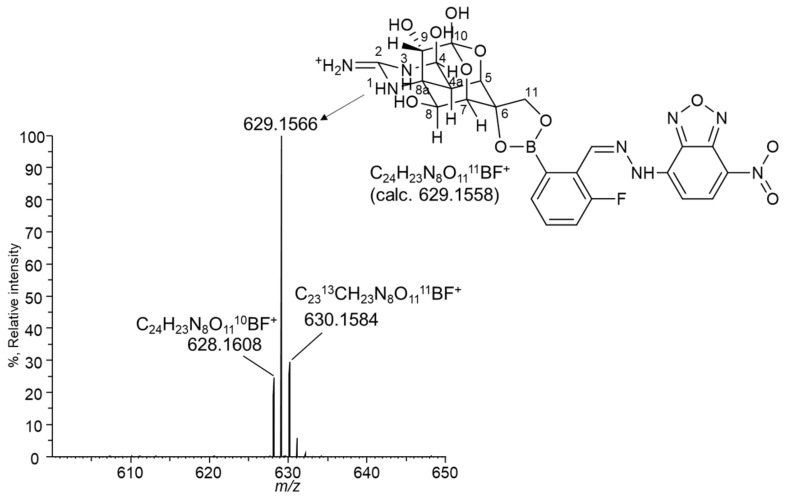
The full mass spectrum of a derivatization product of TTX and NBD-H-DAB (NBD-H-PBA) recorded using LC-HRMS.

**Figure 4:**
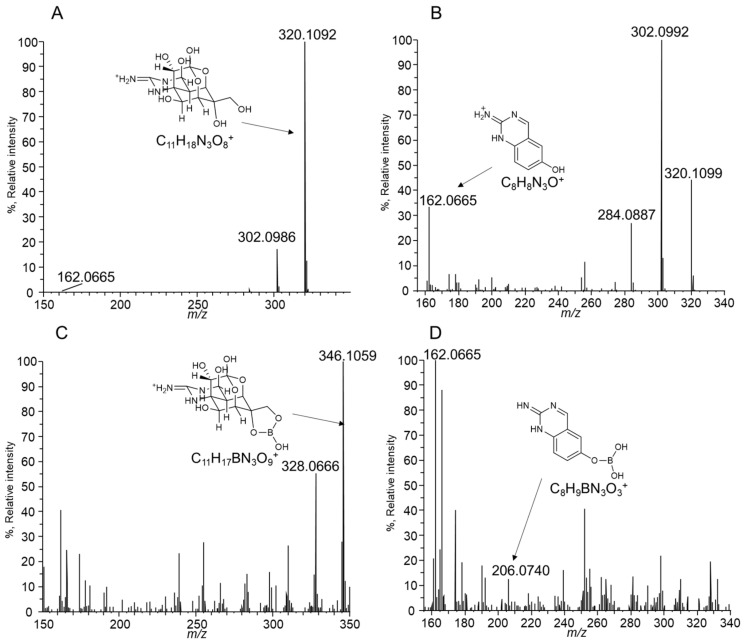
LC-HRMS product ion spectra of the TTX (derived from *m*/*z* 320.1088 as a precursor ion, (**A**,**B**)) and derivatization products of TTX and NBD-H-PBA (derived from *m*/*z* 629.1558 as a precursor ion, (**C**,**D**)). The collision energies were set at 30 eV (**A**,**C**) and 50 eV (**B**,**D**).

The corresponding peak was collected and applied for a high-resolution MS (HRMS) analysis to confirm that the suspected reaction was completed (Figure 3). The LC-HRMS of the derivatized product showed a peak corresponding to [M + H]^+^ (*m*/*z* 629. 1566), and it matched the expected *m*/*z* of the derivatized TTX (*m*/*z* 629. 1558). A comparison of the fragment ions of TTX (Figure 4A) and the derivatized TTX (Figure 4C) showed that the derivatized TTX at CE 30 eV provided a unique fragment (*m*/*z* 346. 1059) in which the boronic acid moiety was bonded to TTX (Figure 4C). At CE 50 eV, a *m*/*z* 206. 0740 fragment ion was detected in the derivatized TTX (Figure 4D), corresponding to the TTX-specific *m*/*z* 162.0665 fragmention (Figure 4B) esterified with boronic acid [25]. These observations confirmed that the boronic acid moiety of NBD-H-DAB was esterified with the 6, 11-diols of TTX. The absorption maxima of NBD-H-DAB (503 nm) and derivatized TTX (508 nm) were approximately equivalent, which was also the evidence for the determination of the derivatized TTX structure (Figure S3A,B).

### 2.4. Analysis of Matrix Sample

**Figure 5:**
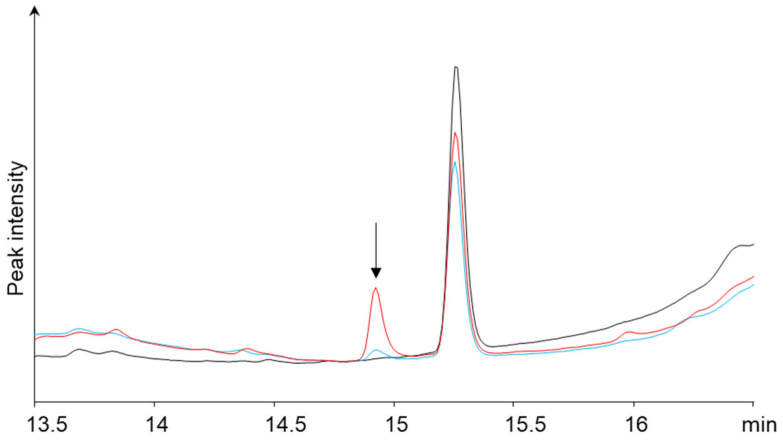
HPLC-UV chromatograms obtained after the derivatization reaction with NBD-H-DAB (NBD-H-PBA) of *Takifugu rubripes* flesh spiked with 0 (black), 2 mg/kg (blue), and 10 mg/kg (red) of TTX.

For the matrix sample analysis, TTX-free flesh specimens of *Takifugu rubripes* spiked with TTX (0, 2, and 10 mg/kg) were extracted and purified as described below (Section 4.4). The resulting extract solution was reacted with NBD-H-DAB and analyzed using RP HPLC-UV. As a result, TTX was derivatized and could be detected at 2 mg/kg (10 mouse unit (MU)/g) (Figure 5). Phenylboronic acid is known to react with sugars [31], but in this study, no peaks corresponding to derivatized products with sugars were identified. Thus, it is suspected that the bulky structure at the reaction site of the NBD-H-PBA might enhance the selectivity of counter compounds such as TTX.

The limit of detection for this analytical method was 0.06 mg/kg (calc. signal-to-noise ratio: 3 in matrix sample), which is sufficient for the analysis of TTX food poisoning levels.

## 3. Conclusions

We developed a novel pre-labeled derivatization reagent that could be used in the analysis of TTX via RP HPLC-UV. The derivatization reagent NBD-H-DAB was designed to have moderate hydrophobicity, UV absorption at long wavelengths, and excellent reactivity with 1,2-diols. Our NBD-H-DAB was rapidly converted to NBD-H-PBA in the reaction solution used and reacted with the 6,11-diols of TTX under weakly acidic conditions (pH 5). Moreover, the derivatized TTX could be analyzed using RP HPLC (510 nm). We were able to detect 10 MU/g of TTX in the spiked fish flesh samples using this analytical method, and we believe that it could present a new alternative to the MBA method. Our method does not require a mass spectrometer or the setup of any other equipment for post-column derivatization. Thus, it is a practical analytical method that can be performed if only reagents and an HPLC-UV are available. To the best of our knowledge, this study details the first TTX pre-labeled derivatization–HPLC method and shows that derivatization is useful for TTX analyses.

## 4. Materials and Methods

### 4.1. Reagents and Materials

The fish specimens used were cultured pufferfish (*Takifugu rubripes*) purchased from a fish retailer in Japan. 4-chloro-7-nitro-2,1,3-benzoxadiazole (NBD-Cl) was purchased from Sigma Aldrich (St. Louis, MO, USA). Acetic acid, sodium acetate, and TTX were obtained from FUJIFILM Wako Pure Chemical Corporation (Osaka, Japan). Hydrazine monohydrate and FFPBA were purchased from Tokyo Chemical Industry (Tokyo, Japan), and all aqueous solutions were prepared using ultrapure water that was purified with a Milli-Q Gradient-A10 system (Merck KGaA, Darmstadt, Germany). All other organic solvents and reagents were of HPLC grade.

### 4.2. Synthesis of NBD-H-DAB

For the synthesis of NBD-H-DAB, 50 mL 1% hydrazine (MeOH) was mixed with an NBD-Cl solution (500 mg in 50 mL of chloroform) and stirred for 60 min. The solution was filtered (ADVANTEC Filter Paper No.5C) and washed with chloroform to obtain NBD-H as a brown solid on the filter. A resulting NBD-H and FFPBA (90 mg) were dissolved with methanol (100 mL) and left to stand for 3 h at 40 °C, then evaporated to dry in vacuo. The residue was suspended in ethyl acetate (200 mL) and washed with 200 mL of 100 mM HCl and brine to remove any remaining reagents. Then, anhydrous sodium sulfate was added to the organic solution to achieve dryness, and the solution was then filtered and concentrated. The residue was subjected to C18 column chromatography (HCOOH-H_2_O-MeCN) to obtain purified NBD-H-DAB (formate) as a red solid. ^1^H NMR (600 MHz, DMSO-*d_6_*): δ 8.78 (s, 1H), 8.48 (d, *J* = 8.8 Hz, 1H), 8.06 (s, 2H), 7.44–7.40 (m, 1H), 7.29 (d, *J* = 8.8 Hz, 1H), 7.20–7.17 (m, 2H) (Figure S1). LC-HRMS (electrospray ionization) calc. for C_13_H_8_O_4_N_5_BF [M + H]^+^: *m*/*z* 328.0647, found 328.0645 (Figure S2). The ^13^C NMR spectrum is not reported as the signal intensity was too weak due to the combined effect of ^19^F splitting and the adjacent ^11^B [32]. UV spectra of the NBD-H-DAB are shown in Figure S3A.

### 4.3. Confirmation of NBD-H-DAB and Derivatized Product of TTX with NBD-H-DAB

TTX containing citric acid as a stabilizer was dissolved in 0.1% acetic acid. To confirm that the derivatization was complete at predicted sites between TTX and NBD-H-PBA, LC-HRMS analysis was carried out with Q-Exactive Plus Orbitrap (Thermo Scientific, Waltham, MA, USA) equipped with the Vanquish UHPLC system (Thermo Scientific, Waltham, MA, USA). The LC-HRMS conditions are listed in Table 1. We used Rs = 17,500 to prevent difficulties in analysis due to the decrease in scan speed. This working resolution assure 2 decimal places. ^1^H NMR spectra were measured on an ECZ 600R spectrometer (JEOL) using deuterated solvents. Chemical shift values were corrected for residual solvent signals as internal standards (DMSO-*d_6_*: 2.50 ppm).

### 4.4. Sample Preparation from Fish Flesh

To extract TTX from the fish specimens and carry out purification, we followed procedures described in previous studies, with some modifications [16,17,33]. Frozen fillet samples were half-thawed under running water, and 5 g of flesh was homogenized with 0.1% acetic acid (*v*/*v*, 5 mL), then heated in boiling water for 10 min. After each sample had cooled at room temperature, it was centrifuged (13,400× *g*, 15 min), and the supernatant (500 µL) was ultra-filtrated with a Pall Nanosep^®^ Centrifugal Device (Omega^TM^ Membrane 10K, Nihon Pall Ltd., Tokyo, Japan). The filtrate and chloroform (500 µL) were mixed well with a vortex, and the organic layer was removed. An SPE cartridge (Supelclean ENVI-Carb 250 mg/3 mL, Supelco, Sigma-Aldrich, St. Louis, MO, USA) was conditioned by first using 3 mL of 1% acetic acid in 20% CH_3_CN and 3 mL of 0.025% ammonium water. Then, 5 µL of 25% ammonium water was added to the extract, and it was loaded onto the cartridge and washed with 700 µL H_2_O. TTX was eluted with 2 mL of 1% acetic acid in 20% CH_3_CN. The eluate was dried under reduced pressure, and the residue was dissolved in 100 µL of a 100 mM sodium acetate buffer (pH 5.0) before being used in the derivatization with NBD-H-DAB.

### 4.5. Derivatization Procedure

The sample solution was mixed with 1000 µL of 2 mg/mL NBD-H-DAB in THF, tightly sealed, and left at 40 °C for 120 min for the derivatization reaction. The resulting product was dried under a N_2_ stream and dissolved with 400 µL of CH_3_OH. The reaction mixture was filtered with using a single STEP Filter vial (0.2 µm PTFE, Thomson Instrument Company, Oceanside, CA, USA) and a 3 µL aliquot of the solution was injected into the HPLC system.

### 4.6. Instrumentation and LC–UV Conditions

LC analysis was performed using a Nexera HPLC system (Shimadzu corporation, Kyoto, Japan) equipped with a system controller (SCL-40), a binary solvent delivery system (LC-40B XR), an autosampler (SIL-40C XR), a column heater oven (CTO-40C), and a photo diode array detector (SPD-M30A, high sensitivity flow cell). The samples were separated with a Mightysil RP-18 PA (150 mm × 4.6 mm i.d., 2.5 µm, Kanto Chemical Co. Inc., Tokyo, Japan). Solvents A (5 mM ammonium formate in 0.1% (*v*/*v*) formic acid) and B (0.1:99.9 (*v*/*v*) mixture of formic acid and 5 mM ammonium formate in 90% (*v*/*v*) CH_3_CN) were used as mobile phases for gradient elution. The flow rate was 0.8 mL/min. The concentration of mobile phase B (%) was maintained at 15% for 0–5 min, then changed linearly from 15 to 70% for 5–20 min and were held at 100% for 20–25 min, and finally held at 15% for 25–30 min. The column oven temperature was set at 25 °C, and the injection volume was 3 µL. UV acquisition was performed at 510 nm (Figure S3B).

## Figures and Tables

**Table 1:** LC-HRMS conditions.

HPLC	Equipment	Vanquish UHPLC System (Thermo Scientific)
	Column	Zorbax Eclipse Plus C18 (1.8 µm, 2.1 × 50 mm, Agilent Technologies, Santa Clara, CA, USA)
	Column temperature	40 °C
	Injection volume	5 µL
	Solvent A	5 mM Ammonium formate in 0.1% Formic acid
	Solvent B	Formic acid and 5 mM ammonium formate in 90% (*v*/*v*) CH_3_CN (0.1:99.9, *v*/*v*)
	Flow rate	0.4 mL/min
	Gradient condition	0–5 min: 10% B, 5–10 min: 10 → 100% B, 10–15 min → 100% B, 15–20 min: 10% B
MS	Equipment	Q-Exactive Plus Orbitrap (Thermo Scientific)
	Ionization	heated electrospray ionization (HESI)
	Spray voltage	3.5 kV
	Auxiliary gas	450 °C, 15 L/min
	Sheath gas	50 L/min
	Capillary temperature	300 °C
	Collision energy	30 and 50 eV
	Resolution	17,500
	AGC target	3e^6^
	Maximum IT	200 ms
	Scan ranges	*m*/*z* 100–1000
MS/MS	Spray voltage	3.5 kV
	Auxiliary gas	450 °C, 15 L/min
	Sheath gas	50 L/min
	Capillary temperature	300 °C
	Collision energy	30 and 50 eV
	Resolution	17,500
	AGC target	2e^5^
	Maximum IT	100 ms
	Precursor	TTX: 320.10884, derivatized TTX: 629.15579

## Data Availability

The data presented in this study are available on request from the corresponding authors.

## Supplementary Materials

The following supporting information can be downloaded at https://www.mdpi.com/article/10.3390/toxins16060260/s1: Figure S1: The ^1^H NMR spectrum (DMSO-*d_6_*, 600 MHz) of synthesized NBD-H-DAB; Figure S2: The LC-HRMS of NBD-H-DAB; Figure S3: The UV spectra of NBD-H-DAB (A) and derivatized TTX with NBD-H-PBA (B); Figure S4: Comparison of relative peak areas of derivatized TTX (ratio to maximum peak) under various conditions (A: pH, B: THF concentration, C: NBD-H-DAB D: reaction temperature and time) to optimize derivatization reaction conditions.
