# Supplementary material for: Tetrodotoxin Derivatization with a Newly Designed Boron Reagent Leads to Conventional Reversed-Phase Liquid Chromatography
Source: Toxins (Basel). 2024 Jun 4;16(6):260. doi: 10.3390/toxins16060260 (PMC11209320; doi:10.3390/toxins16060260)
Supplement: Supplementary file 1 [file toxins-16-00260-s001.zip › toxins-3028661-supplementary.pdf]

Supplementary Materials

Table of contents

Figure S1. The ^{1}H NMR spectrum (DMSO-d_6, 600 MHz) of synthesized NBD-H-DAB.
Figure S2. The LC-HRMS of NBD-H-DAB.
Figure S3. The UV spectra of NBD-H-DAB (A) and derivatized TTX with NBD-H-PBA (B).
Figure S4. Comparison of relative peak areas of derivatized TTX (ratio to maximum peak) under various conditions (A: pH, B: THF concentration, C: NBD-H-DAB D: reaction temperature and time) to optimize derivatization reaction conditions.

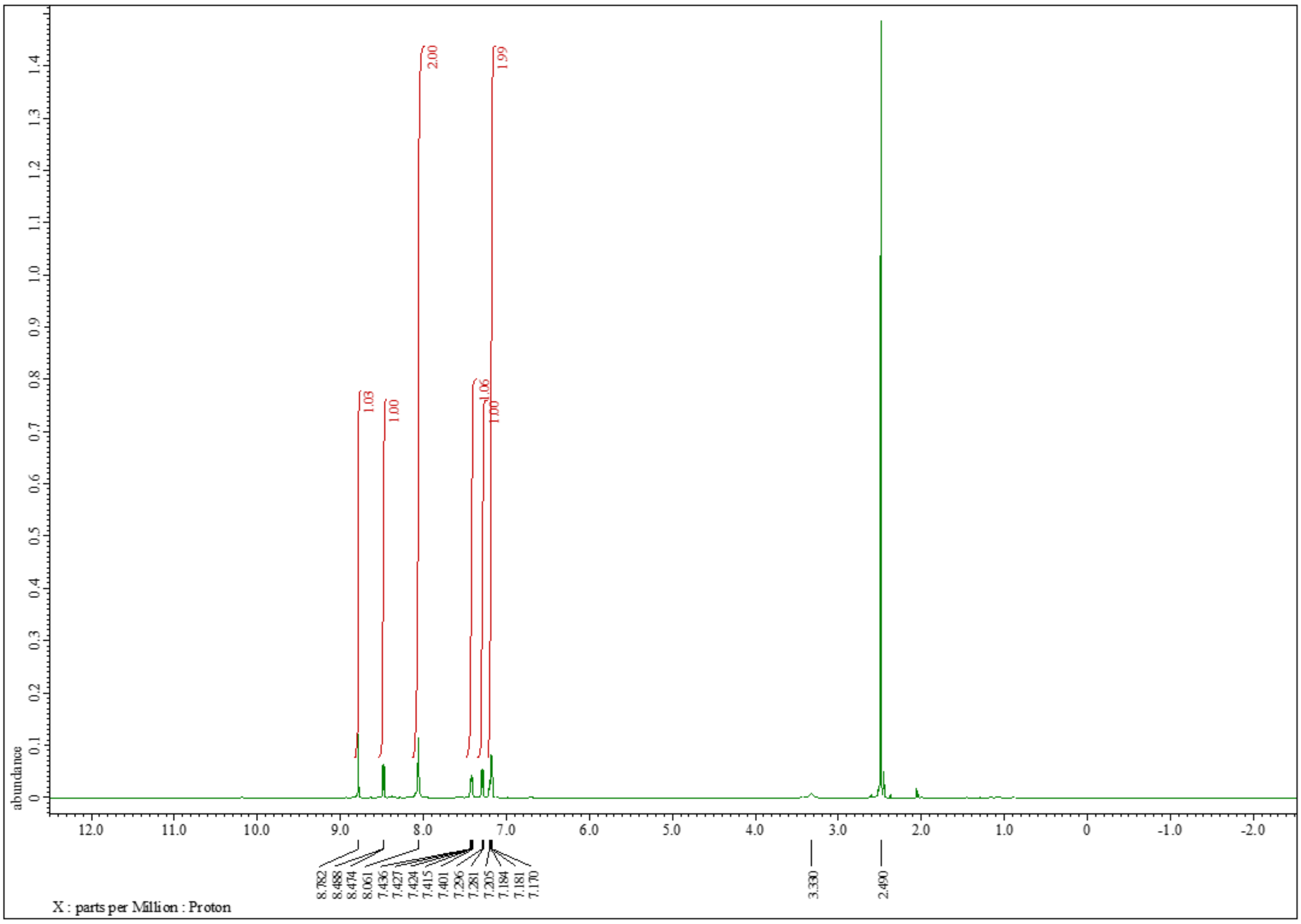

Figure S1. The ^1H NMR spectrum (DMSO-d_6, 600 MHz) of synthesized NBD-H-DAB.

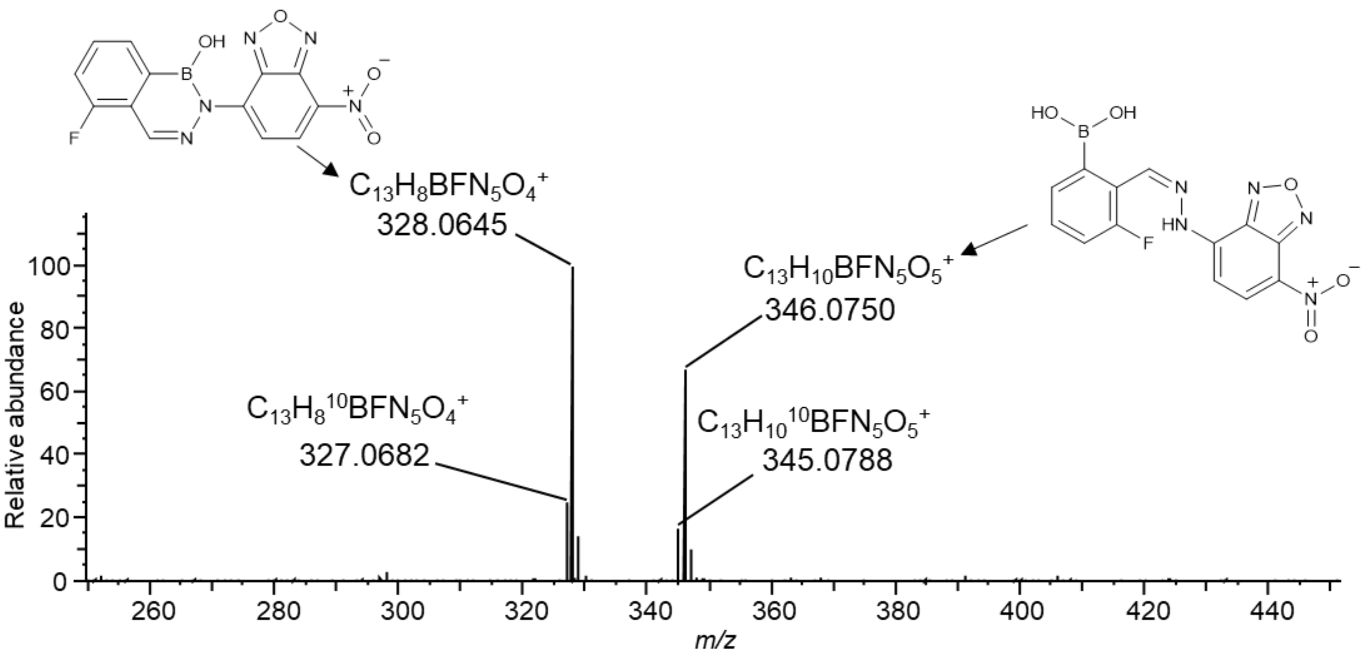

Figure S2. The LC-HRMS of NBD-H-DAB. NBD-H-DAB was partially converted to NBD-H-PBA by hydrolysis in the aqueous mobile phase.

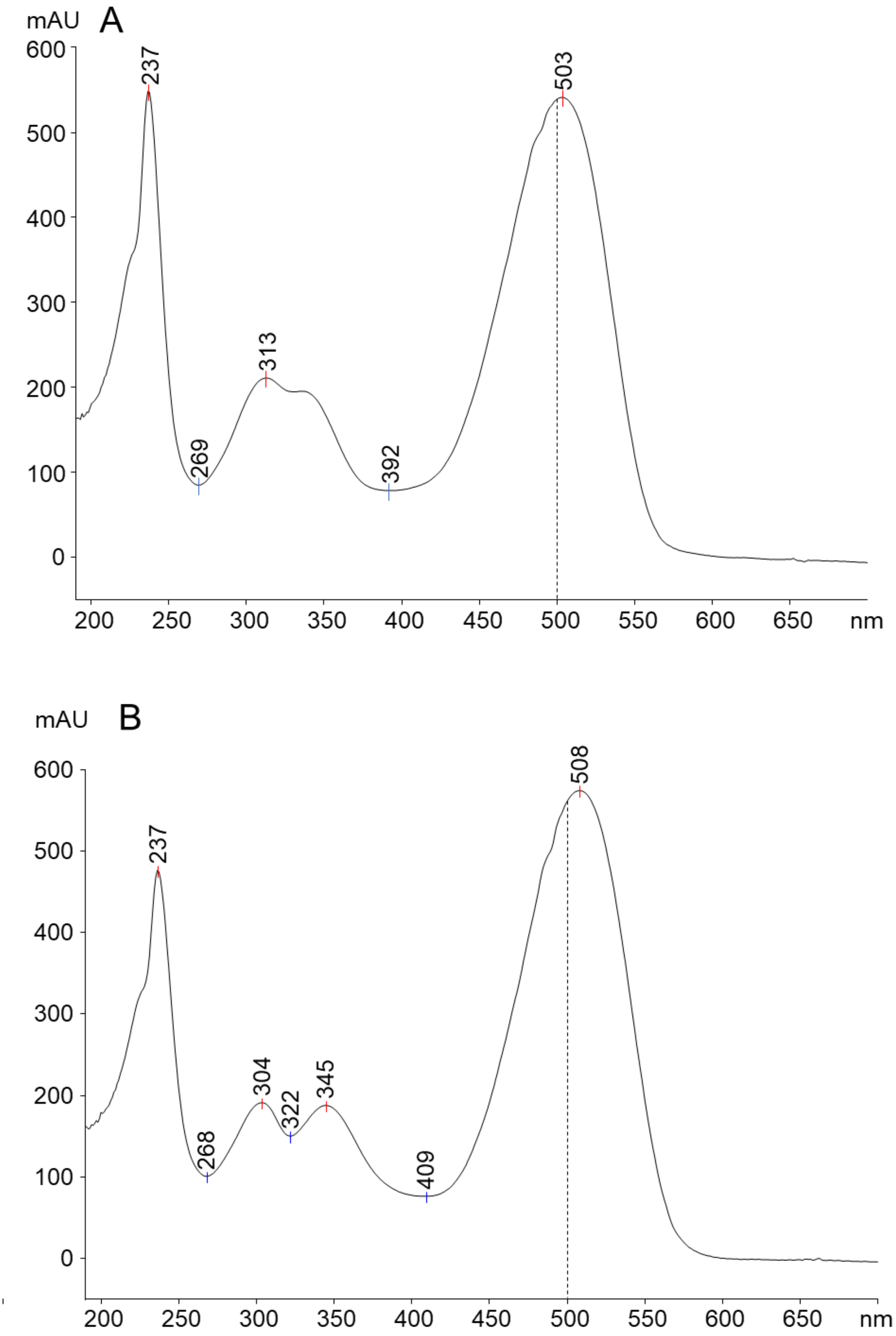

Figure S3. The UV spectra of NBD-H-DAB (A) and derivatized TTX with NBD-H-PBA (B).

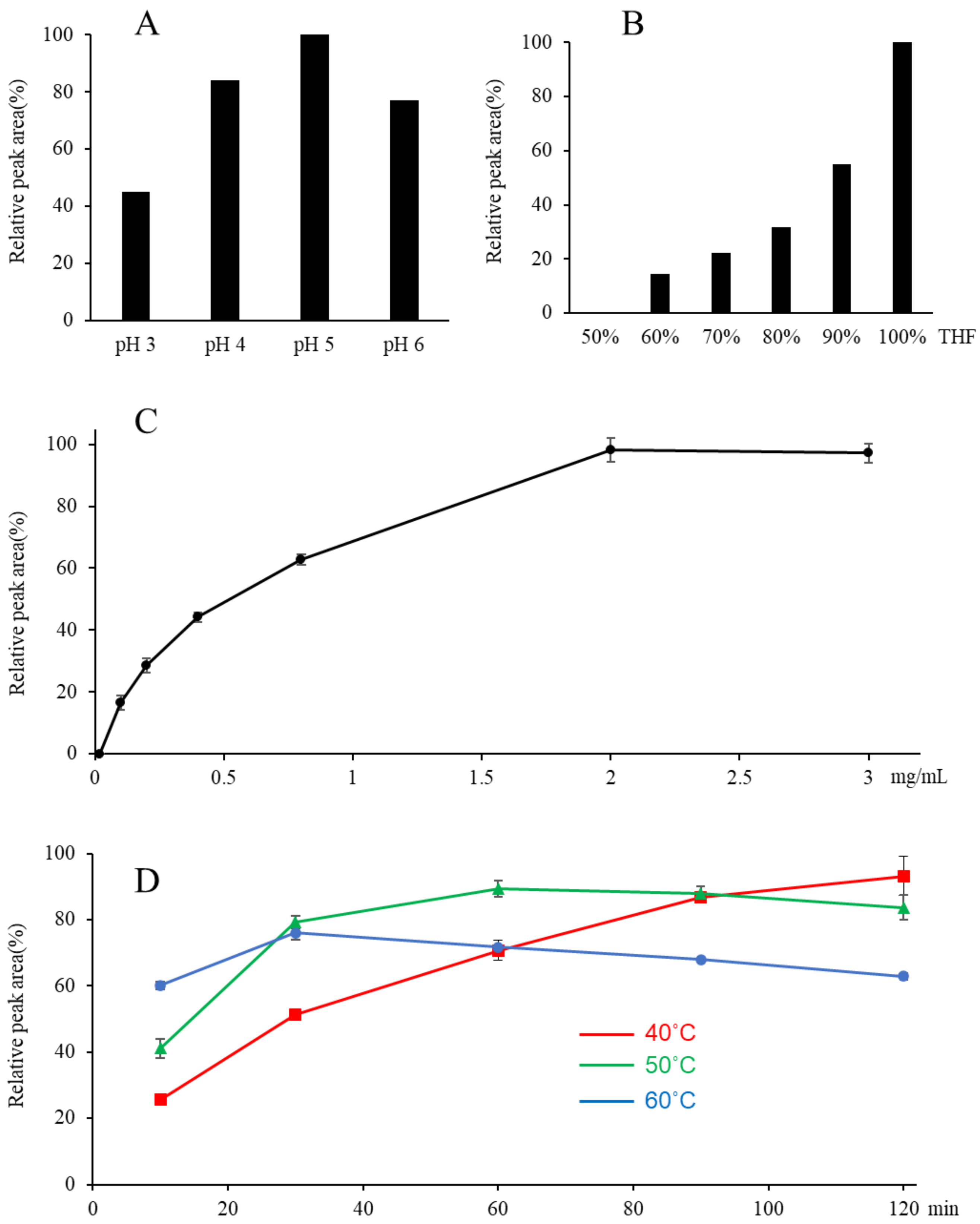

Figure S4. Comparison of relative peak areas of derivatized TTX (ratio to maximum peak) under various conditions (A: pH, B: THF concentration, C: NBD-H-DAB D: reaction temperature and time) to optimize derivatization reaction conditions.
